# Effects of Local Habitat Variation on the Behavioral Ecology of Two Sympatric Groups of Brown Howler Monkey (*Alouatta clamitans*)

**DOI:** 10.1371/journal.pone.0129789

**Published:** 2015-07-06

**Authors:** Linda Jung, Italo Mourthe, Carlos E. V. Grelle, Karen B. Strier, Jean P. Boubli

**Affiliations:** 1 Thuringian State Institute of Agriculture, Jena, Germany; 2 Faculdade de Biociências, Pontifícia Universidade Católica do Rio Grande do Sul, Porto Alegre, Brazil; 3 Departamento de Ecologia, Universidade Federal do Rio de Janeiro, Rio de Janeiro, Brazil; 4 Department of Anthropology, University of Wisconsin, Madison, Wisconsin, United States of America; 5 School of Environment and Life Sciences, University of Salford, Salford, United Kingdom; University of Illinois at Urbana-Champaign, UNITED STATES

## Abstract

Although the brown howler monkey (*Alouatta clamitans*) is a relatively well-studied Neotropical primate, its behavioral and dietary flexibility at the intra-population level remains poorly documented. This study presents data collected on the behavior and ecology of two closely located groups of brown howlers during the same period at the RPPN Feliciano Miguel Abdala in southeastern Brazil. One group occupied a primary valley habitat, henceforth the Valley Group (VG), and the other group occupied a regenerating hillside habitat, the Hill Group (HG). We hypothesized differences in the behavior and ecological parameters between these sympatric groups due to the predicted harsher conditions on the hillside, compared to the valley. We measured several habitat parameters within the home range of both groups and collected data on the activity budget, diet and day range lengths, from August to November 2005, between dawn and dusk. In total, behavioral data were collected for 26 (318 h) and 28 (308 h) sampling days for VG and HG, respectively. As we predicted, HG spent significantly more time feeding and consumed less fruit and more leaves than VG, consistent with our finding that the hillside habitat was of lower quality. However, HG also spent less time resting and more time travelling than VG, suggesting that the monkeys had to expend more time and energy to obtain high-energy foods, such as fruits and flowers that were more widely spaced in their hill habitat. Our results revealed that different locations in this forest vary in quality and raise the question of how different groups secure their home ranges. Fine-grained comparisons such as this are important to prioritize conservation and management areas within a reserve.

## Introduction

From the point of view of a primate, rainforest habitats are not homogeneous places. Fine-grained variations in environmental conditions at the scale of a single study site are expected due to variation in elevation (topography) and associated water table, steepness of terrain, soil nutrient gradients among other factors [1]. In turn, these environmental variables will affect the structure and composition of local plant communities contributing to the heterogeneity in local ecological resources and conditions available to primates [2]. Primates with large home ranges negotiate such fine-grained heterogeneity by traveling across the landscape seeking out patches of high quality habitat (e.g., [3,4,5]). Species with small home ranges however, may need to restrict their ranges to areas of higher quality habitats if they are to find their preferred foods while avoiding competitors and predators (e.g., [4,6,7,8]).

In situations of high population densities and with limited opportunities for dispersal, as is the case in forest fragments with low predator abundances some primate groups might be pushed to lower quality parts of the forest [9]. We expect animals inhabiting such lower quality habitats to be under greater ecological stress to meet their daily nutritional requirements, i.e., having to travel further each day to find preferred high quality foods (energy maximization) or having to rest more to save energy while eating lower quality foods (time minimizing) [3,10,11], and consuming a limited set of resources, including less fruit and more foliage [12,13].

Howler monkeys (*Alouatta* spp.) are folivorous-frugivorous, arboreal primates that generally do not come to ground to feed, and rely on large trees of certain species. These primates often rely heavily on mature and young foliage along the annual cycle and have a number of adaptations to deal with this leaf-based diet such as an extensive hindgut area and slow passage rates [14–18]. The brown howler monkey (*Alouatta clamitans*) is a mid-sized howler monkey with a wide geographical distribution in the Atlantic forests of Brazil, and northeastern Argentina [19,20]. This species is found at high density (29 ind./km^2^) in the 1,000 ha Atlantic Forest fragment of the RPPN-FMA, in Caratinga, Brazil. At this site, howlers live in small groups (~5–6 individuals) and in small home ranges [21,22].

Due to the hilly terrain and recent history of human disturbances (agriculture, fires, logging), the forest in Caratinga is considerably heterogeneous [23]. There are open patches dominated by bracken, young secondary forest growing on old coffee plantations, grasses and dirt roads. The structure, floristic composition and amount of herbaceous vegetation also vary in significant ways between the three main landscape features of the site: valleys, hillsides and hilltops [23].

We wanted to determine if habitats we perceived as lower quality for primates, i.e., hilltop and hillsides that presented lower tree species diversity, greater number of deciduous trees, lower structural complexity (fewer big trees, less connectivity and fewer canopy layers and less ground vegetation) and a recent history of human disturbance [23], were in fact of lower quality to howlers. Such information is important for better understanding howler habitat preferences and requirements, which ultimately is invaluable information for the management and zoning of priority areas for conservation within this reserve.

We chose to follow two howler monkey groups of similar size and composition; one in a valley bottom habitat that we considered high quality, henceforth Valley Group (VG) and another on a hillside next to the VG, a lower quality habitat, henceforth Hillside Group (HG). The VG experienced high local humidity characterized by mature forest with few deciduous trees whereas the HG experienced a more disturbed 40-year-old secondary forest at a hillside location with drier conditions and many deciduous tree species [23]. We compared diet, time budget, and travel distances of these two closely located groups of howler monkeys inhabiting these contrasting habitats.

Primary consumers are challenged with the highly variable nutritional content and spatiotemporal distribution of their potential foods [3,4,24–26]. In a folivorous-frugivorous diet such as that of the howler monkeys, increased leaf consumption is hypothesized to lead to an increased feeding time because leaves are low in energy and more food is needed to achieve satiation [3,27,28]. Due to its low energy content, a leaf-based diet is often associated with energy-saving strategies i.e., a greater amount of time spent inactive during the day and reduced travel time (time-minimizing-strategy) [3,10,29–31]. Consequently, increasing leaf consumption leads to shorter travel distances while increasing fruit consumption, a source of high energy, has the opposite effect. Although howlers are generally thought to have a leaf-dominated diet and to be energy-limited, some studies have indicated that they are not [32,33]. We hypothesized that the HG would be under greater ecological stress due to the lower quality of this habitat. Thus, we predicted that 1) HG howlers would consume less fruit and more mature leaves than the VG howlers and; 2) due to the energetically poorer diet, the HG would devote more time to feeding and resting and less time to travelling (time-minimizing-strategy) than the VG.

## Methods

### Ethics statement

We declare that this research was observational only and that all observations were carried out in accordance with the current laws of Brazil. Our research protocols were approved by the administration of the RPPN Feliciano Miguel Abdala and adhered to the Code of Best Practices for Field Primatology of the American Society of Primatologists and International Primatological Society (www.asp.org/resources/docs/Code%20of_Best_Practices%20Oct%202014.pdf).

### Site and species

The study was conducted at the RPPN Feliciano Miguel Abdala (RPPN-FMA), a privately owned reserve located in the state of Minas Gerais, southeastern Brazil (19°50’S, 41°50’N; S1 Fig). The area is hilly with varying altitudes between 400 to 680m [34]. The RPPN-FMA comprises an area of approximately 1,000 ha of Atlantic forest, which represents an important forest remnant in a highly fragmented forest landscape. The region is characterized by a temperate climate with a strongly seasonal pattern of hot rain-laden summers (rainy season) and dry winters (dry season), as described after Köppen (Cwa) [35]. More than 80% of the annual rainfall occurs during the rainy season, which lasts from November to April. The annual temperature and rainfall average 20.6 ± 2.9°C (2002–2004) and 1,119.8 ± 262.75 mm (1986–2001, updated from [36],) respectively.

We selected two study locations in the Jaó Valley, the northern part of the reserve (S1 Fig), lying in close vicinity of each other (ca. 300m), each one inhabited by one group of brown howlers. Although closely located, the study groups used non-overlapping areas (S1 Fig). The first site comprised a valley and surrounding hills. The valley was characterized by evergreen forest with a small number of deciduous tree species (i.e. those that lost their leaves during the dry season). The second study site was located along a hillside that had been used as a coffee plantation in the past. The vegetation consisted of a 40-year-old secondary forest with a great number of deciduous trees. Boubli et al. [23] contrasted the structure and floristic composition of valley and hill forest habitats at RPPN-FMA. They found valley habitats to be richer in tree species (119 vs. 81 species for trees ≥ 10 cm diameter at breast height; DBH) and with larger trees (basal area per tree was approximately double that of the hill habitats). Both habitats shared only 39 species of tree.

The study area comprising the valley will be referred to as the valley site from here on, although it also includes hill-habitat, and the observed howler group is named VG. Likewise, HG is the howler group inhabiting the hillside site. The VG consisted of six animals, i.e. one adult and one subadult male, two adult females, one subadult female and one juvenile. The HG comprised five individuals; one adult and one subadult male, and two adult females, one of them with a dependent infant.

### Microhabitat characterization

To characterize the microhabitats used by the study groups, we used a modification of Boubli et al. [37] and August [38] methods. Habitat structural attributes were assessed by an observer positioned at the center of fifty imaginary 100 m^2^ quadrats located within the range of each group. The location of the quadrats was determined as follows: 50 points were chosen at 20 m distance along walking trails crossing the study habitats. From each point we walked 10 m perpendicular to the trail into the forest, this new point being the middle of each 100-m^2^ quadrats within which the following variables were assessed: number of emerging trees (trees that emerged above the canopy), number of canopy layers, canopy height (height of majority of trees), canopy density (density of the canopies of all trees), connectivity (connection of all layers that are important for monkey travelling, regarding the connection of the vegetation within the quadrat as well as its connection to the adjacent vegetation in walking direction), canopy continuity (opposite to canopy fragmentation), and density of lianas. Number of emergent trees, number of layers, and canopy height were estimated directly. All other variables were evaluated using a subjective scale varying from 0–4 (0 = absent, 1 = 1–25%, 2 = 26–50%, 3 = 51–75%, 4 = 76–100%) [37]. Only a single observer (LJ) assessed these variables to avoid inter-observer biases.

To estimate tree density we used the point-quadrant method [39]. At each point we measured the diameter at breast height (DBH) and the distance from each tree to the central point for the nearest tree ≥10 cm DBH in each quarter and their identification whenever possible. Several trees in our samples had multiple trunks, in which case we considered the quadratic DBH that is the square root of all summed squared DBHs (i.e., √(DBHi^2^ + DBHi^2^)) [23]. The diversity and evenness of trees in the valley and hillside areas were estimated using Shannon Index (H’), calculated in the natural log basis, and Pielou Index (J) [40]. H’ is a quantitative measurement of diversity that accounts for the number of species present and their relative abundances. The higher the H’ the higher the diversity. J is derived from H’ and represents the uniformity in the distribution of the individuals between the species in the sampled assemblage, varying from 0 to 1 (maximal uniformity).

### Behavioral data collection

Both groups were already partially habituated to human presence due to previous research work in the area [41] and the presence of local people living close by. However, prior to systematic data collection, a brief habituation period of 6 to 8 days was conducted to familiarize the howler groups with the presence of the observer (LJ). Behavioral data were collected from August to November 2005, using the Scan Sampling method [42], with a 5-min scan conducted at 15 min intervals, starting between 5:15–6:15 a.m. and finishing when the howler monkeys entered their sleeping tree in the evening, i.e., between 5:15–6:00 p.m. each sampling day. This method allowed us to obtain data from all individuals per scan except in cases when some individuals were out of sight [42]. Data collection of a group was preceded by a search period, which generally took about 2–4 hours and was carried out in the morning. Once one of the study groups was found, data were collected for a maximum of eight consecutive and complete (8–12h) days before switching to the other group. This period was termed sample session. The time between two sample sessions never comprised more than 6 days. A total of three sample sessions was conducted for each group during 26 (318 h) and 28 (308 h) sampling days for VG and HG, respectively.

During each scan, the first activity state lasting at least 5 s for each individual sighted was recorded. Behavioral records were classified into six categories: resting, moving (within the same tree), travelling (between trees), feeding (inspection of food, bringing it to the mouth, chewing and swallowing), social interaction (grooming, social play behaviors), and others (e.g. defecation, urination, social vocalization, copulation). When animals were observed feeding, an effort was made to identify and record the plant part ingested (fruit, mature/immature leaf, mature/immature stem, flower) and its origin (liana or tree). Food sources were later identified to the lowest taxonomic level possible.

The percentage of each activity of the total time budget was calculated using the proportional method [43]: proportions of each activity were first calculated per scan and then averaged over all scans per day, all days, and finally months of the study. Dietary data were treated similarly but only feeding scans were considered in the calculation. This way, percentage of feeding time spent on different food items was determined, which served to quantify the relative importance of each food item in the diet. We used Spearman rank correlation coefficients between percentage of time spent by each group in different behavioral activities and food items consumed.

The location of the study group during every scan sample and the location of food trees were recorded with GPS (Garmim GPS 76) and subsequently plotted in a map (S1 Fig). Daily travel distances were estimated by summing the distance between consecutive group location records made throughout the day. Total home range used by each group was measured by using the Hawth’s Tools Animal Movement extention of ArcGIS 9.1. We used the Minimum Convex Polygon option to calculate the areas of the home ranges. The dependent infant in HG was not included in behavioral sampling because it was generally carried by its mother and hidden from view during most of the time. Analyses were carried out in R [44]. As most data were not normally distributed, we used the nonparametric statistics, Spearman rank correlation (r_s_) and Wilcoxon rank sum test (W) to correlate and compare data, respectively. Significance level was set at 0.05.

## Results

### Microhabitat comparison

In the hillside area, 3.5% of trees were > 40 cm DBH with the largest tree measuring 60.5 cm, whereas in the valley, 8% of the trees were above 40 cm DBH with the largest tree measuring 155 cm DBH. The density of trees was the same for the valley and hillside habitats (0.08 trees/m^2^). In total, 74% of all 400 trees measured were identified. In the valley habitat, there were at least 43 species versus at least 29 species in the hillside habitat. Both habitats shared at least 19 of the identified species. The valley habitat was more diverse and even than the hill habitat (H’ = 3.47; J = 0.92 and H’ = 2.39; J = 0.71). In the hill habitat, *Dalbergia nigra* represented at least 30% (n = 62) of all trees sampled, explaining the low evenness obtained.

The VG occupied an area with a more connected upper canopy layer, relatively denser shrub layer, and higher number of layers and emergent trees than the area used by the HG (Table 1). Although average DBH was not significantly different between the two habitats, there was a higher percentage of trees with multiple trunks in the hillside (21%) versus the valley (4%). Trees with multiple trunks are typical in young secondary forests at our study site [21]. None of the other variables measured here were significantly different between habitats.

**Table 1:** Comparison of habitat characteristics between the home ranges of the Valley (VG) and Hill Group (HG) using Wilcoxon rank sum test. See methods for detailed description of variables.

Microhabitat variables	W	*P*	VG (*n* = 50) Mean ± SE	HG (*n* = 50) Mean ± SE
Canopy density	1054	0.14	2.4 ± 0.10	2.2 ± 0.10
Canopy height	1124	0.38	15.2 ± 0.62	14.5 ± 0.56
DBH	10745	0.39	21.9 ± 1.14	19.2 ± 0.59
Tree height	10318	0.22	15.5 ± 0.58	14.0 ± 0.34
Number of emergents	879	< 0.001	0.4 ± 0.09	0.1 ± 0.07
Number of layers	676	< 0.001	1.1 ± 0.13	0.3 ± 0.09
Connectivity	539	< 0.001	2.9 ± 0.13	2.0 ± 0.11
Continuity	881	< 0.01	2.4 ± 0.13	1.9 ± 0.11
Density of mid-store	1044	0.12	2.2 ± 0.12	2.0 ± 0.9
Density of lianas	1387	0.32	2.1 ± 0.14	2.3 ± 0.12
Density of shrub	969	0.03	2.6 ± 0.12	2.2 ± 0.11
Percentage of bare ground	837	< 0.01	3.0 ± 0.11	2.5 ± 0.10

### Howler monkey behavior and ecology

In total, 2,408 scan samples were collected, 1,274 for the VG and 1,234 for the HG. Howler monkeys fed on at least 58 plant species belonging to 24 families (S1 Table). These species represent roughly 27% of the 214 woody plant species found in the study site [23].

VG diet included at least 44 plant species (26 identified trees plus 18 tree and liana morphospecies) as compared to at least 34 species for HG (22 identified trees plus 12 tree and liana morphospecies) (S1 Table). Whereas the VG spread its diet more evenly across all species eaten, HG devoted a disproportionate amount of time to *Apuleia leiocarpa*, spending 23.3% of all feeding time eating leaves (22%) and flowers (3%) from this tree species. The most important species in VG diet in this study was *Ficus sp*., which comprised nearly 16% of the monkeys’ feeding time.

**Fig 1:**
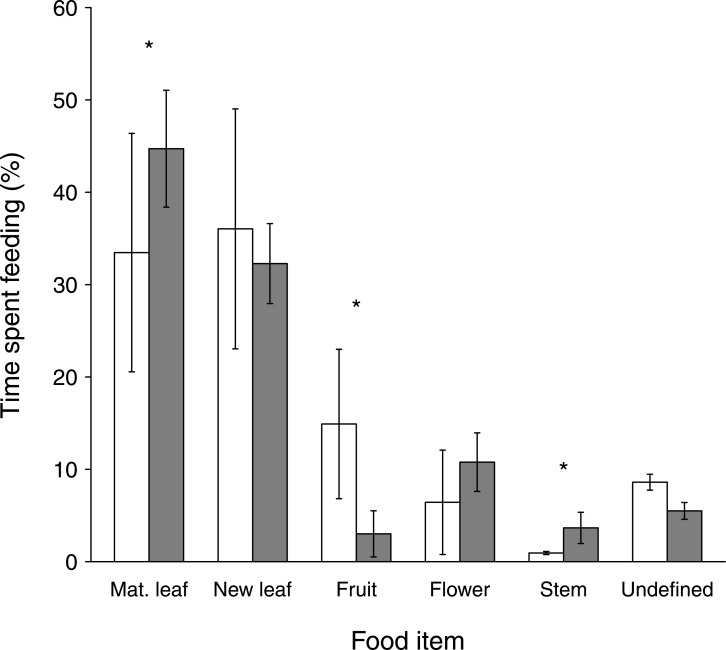
Time spent feeding on different food items in % ± SE for two groups of *Alouatta clamitans* from August to October 2005 at RPPN-FMA, Minas Gerais, Brazil. White bars = Valley Group, grey bars = Hill Group. Asterisks indicate significant differences, as described in the text.

Trees were the most used food source in both groups. The consumption of tree items (68% vs. 78%; W = 268, *p* = 0.10) and liana items (22% vs. 15%; W = 450, *p* = 0.14) did not differ significantly between the VG and HG, respectively. The largest part of feeding time was spent on leaves in both groups (Fig 1: 71% vs. 77%, respectively; W = 266, *p* = 0.09). Feeding time spent on mature leaves was significantly lower (W = 241.5, *p* = 0.04) in the VG (34%) compared to the HG (45%) but the groups did not differ from one another in their consumption of immature leaves (W = 412.5, *p* = 0.41). Fruits and flowers were the second most important food item in the VG and in the HG, respectively. Feeding time on fruit was significantly shorter in the HG compared to the VG (3% vs. 15%, respectively; W = 523, *p* = < 0.01). There was a tendency for higher flower consumption in the VG (11%) than in the HG (6%) (W = 258.5, *p* = 0.05).

**Fig 2:**
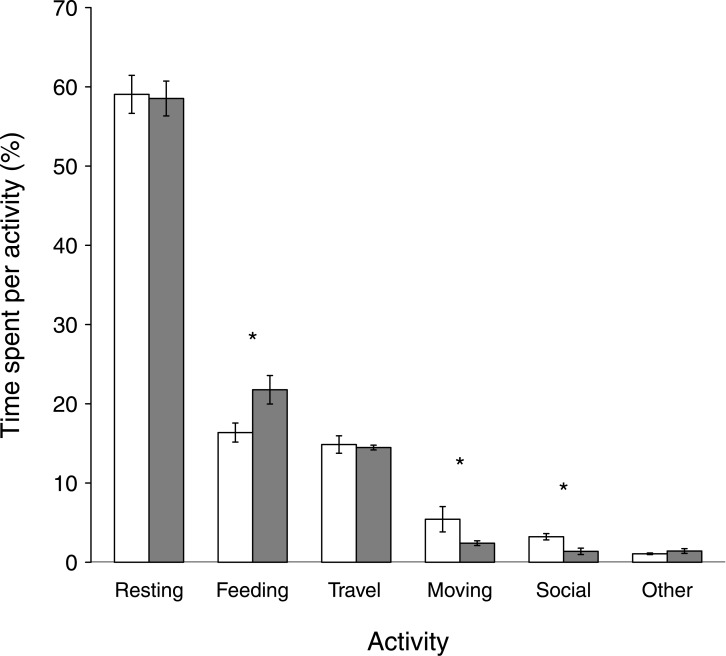
Time spent in different activities in % ± SE for two groups of *Alouatta clamitans* from August to October 2005 at RPPN-FMA, Minas Gerais, Brazil. White bars = Valley Group, grey bars = Hill Group. Asterisks indicate significant differences, as described in the text.

Both groups spent an equal amount of daytime resting (Fig 2: 59%; W = 381.5, *p* = 0.77) and travelling (15%; W = 329.5, *p* = 0.56; Fig 2). Feeding time was significantly lower in the VG, where it contributed 16% to overall time budget, than in the HG (22%; W = 117, *p* < 0.001). In contrast, moving was significantly higher in the VG (5%) than in the HG (2%; W = 585, *p* < 0.001), as was time spent in social interactions (3% vs. 1%, respectively; W = 579, *p*<0.001).

### Correlation between diet and time budget

The percentage of time spent in several behavioral activities by each group was significantly correlated with the time spent consuming different dietary items. These correlations are summarized in Table 2. Feeding time decreased significantly with an increasing intake of fruit and mature foliage in the HG and flowers in the VG. Resting time decreased with the intake of flowers and immature leaves and increased with the consumption of mature leaves in the HG. There was also a tendency for increasing resting time with increasing fruit consumption in HG. In general, no significant correlations were found between travel time and food type consumption. Moving time however, was positively influenced by fruit intake and negatively by the consumption of flowers in the VG. Time spent in social interactions was only significantly influenced by diet in the VG, being negatively correlated with feeding time on mature leaves and positively with immature leaves (Table 2). There was evidence for time budget limitation in both groups, for which resting and feeding time (VG: r_s_ = -0.454, *n* = 28, *p* = 0.02; HG: r_s_ = -0.526, *n* = 26; *p* < 0.01), as well as resting and travel time (VG: r_s_ = -0.39, *n* = 28, *p* = 0.05; HG: r_s_ = -0.68, *n* = 26, *p* < 0.001) were negatively correlated. Resting was also negatively influenced by moving time, but just in the VG (VG: r_s_ = -0.47, *n* = 28, *p* = 0.01; HG: r_s_ = -0.36, *n* = 26, *p* < 0.07).

**Table 2:** Spearman rank correlation coefficients of the relation between percentage of time spent by each group in different behavioral activities and items consumed. Significances are shown in parentheses. Number of samples for VG = 28 and HG = 26.

Activities	Item consumed			
	Fruit	Flowers	Mature leaf	Immature leaf
Feed VG	0.32 (0.10)	-0.48 (0.10)	0.36 (0.06)	-0.33 (0.09)
Feed HG	-0.45 (0.02)	0.26 (0.20)	-0.38 (0.05)	0.14 (0.48)
Move VG	0.54 (< 0.01)	-0.43 (0.02)	-0.07 (0.73)	-0.20 (0.32)
Move HG	-0.0002 (1.00)	0.30 (0.14)	-0.18 (0.39)	0.12 (0.57)
Travel VG	-0.36 (0.06)	0.17 (0.37)	0.20 (0.32)	-0.09 (0.63)
Travel HG	-0.003 (0.99)	0.13 (0.53)	-0.31 (0.12)	0.34 (0.09)
Rest VG	-0.22 (0.27)	0.24 (0.22)	-0.19 (0.34)	0.26 (0.18)
Rest HG	0.37 (0.07)	-0.45 (0.02)	0.67 (< 0.001)	-0.59 (0.001)
Social VG	-0.11 (0.59)	0.03 (0.89)	-0.39 (0.04)	0.50 (< 0.01)
Social HG	0.21 (0.30)	0.36 (0.07)	-0.24 (0.24)	0.30 (0.14)

### Ranging pattern

The home range of VG calculated for the duration of this study was smaller (5.03 ha) than that for the HG (15.80 ha). Total travel distance for the combined three months of the study was longer for the HG than for VG (13,015 m vs. 9,332 m, respectively). The HG travelled significantly longer distances per day than the VG, i.e. 542 ± 41 m vs. 389 ± 62 m, respectively (W = 150, *n* = 24, *p* < 0.01). As expected, daily travel distance was strongly and positively correlated with travel time in both groups (VG: r_s_ = 0.790, *n* = 24, *p* < 0.001; HG: r_s_ = 0.727, *n* = 24, *p* < 0.001). Feeding time decreased significantly with daily travel distance in the VG (r_s_ = -0.583, *n* = 24, *p* < 0.01) and showed the same tendency in the HG (r_s_ = -0.389, *n* = 24, *p* = 0.06). Furthermore, daily travel distance and time spent in social interactions were positively correlated in the HG (r_s_ = 0.527, *n* = 24, *p* < 0.01) but not in VG (r_s_ = -0.07, *n* = 24, *p* = 0.74). Interestingly, only feeding time on flowers correlated significantly and positively with travel distance per day in the VG (r_s_ = 0.471, *n* = 28, *p* = 0.02). Travel distance per day and intake of fruit were not correlated in any group, but showed a tendency for a negative correlation in the VG (r_s_ = -0.352, *n* = 28, *p* = 0.09) and a positive one in the HG (r_s_ = 0.391, *n* = 26, *p* = 0.06).

## Discussion

This study revealed differences in diet, time budget, and travel distance between our two study groups. Although such variation has previously been documented between *A*. *clamitans* populations separated by several hundred kilometers [22,27,30,45,46] and within single *Alouatta* groups in different seasons [22,27,30], such ecological and behavioral differences in contrasting microhabitats in the same area are rarely studied [3].

In accordance with our first prediction, the HG consumed less fruit and more leaves than the VG. The highest fruit intake in the VG occurred during September and was mostly due to several very large fruit trees available in its home range. In particular, a large fig tree was an important source of fruit to the VG during this study. Other authors have emphasized the importance of *Ficus* in the diet of howler monkeys [3,17,47,48] and Serio-Silva et al. [49] suggested that the degree of frugivory in howler monkeys is closely related with fig production; when no figs are present, folivory dominates. At the hill site, only one small fig tree was recorded but it did not produce fruit during the study period. We believe moving time was greater in VG because howlers in this group spent more time foraging within such large fruit tree canopies to find ripe fruits.

In agreement with our second prediction, the HG spent significantly more time feeding than the VG. Although this find is consistent with our results that HG ate more leaves than VG, we found a negative relationship between time spent feeding and the consumption of mature leaves. This is harder to interpret since we expected that, given the lower quality of leaves as compared to fruits in terms of energy sources, HG monkeys had to devote more time to feeding than VG to meet their daily energy demands. In addition, feeding time included the proportion of time the animals spent chewing leaves; a greater proportion of feeding time is required for processing (chewing) highly fibrous leaves than fruit and flowers that require less mastication per quantity ingested. Experiments on captive *A*. *palliata* have shown that twice as much time is required for consuming the same amount of fresh foliage compared to fruit [7]. Our interpretation of this result is that, on days when howlers spent more time eating mature leaves, they required longer resting times in order to digest this food item, which might have influenced the time devoted to feeding as time devoted to the six activities recorded here are all interdependent.

Our prediction that the HG would spend more time resting and less time travelling than the VG, was not confirmed. Both groups devoted the same amount of time to rest and travel. The reasoning behind our initial prediction was that in tropical forests, higher quality foods have been shown to be patchily distributed in space and time [3]. Travel and resting time have been related to food source distribution [3,5,50,51]. Thus, great travel distances, long travel times and consequently less resting time have been associated with patchily distributed fruit, flowers and young leaves, whereas short travel distances, small travel times and more resting with uniformly distributed mature leaves [48,52,53].

We attribute our unexpected result to the marked differences in habitat quality we found between HG and VG. During our study, the VG fed mostly on a few large trees available in their home range that provided the majority of fruits consumed by this group. Sometimes, the group spent almost the entire day feeding on one very large *Ficus sp*. tree, a large food patch that did not require increased travelling to find fruit. Indeed, the effect of shorter day ranges associated with camping out (and thus, resting) at large patches of preferred fruits has previously been described for sympatric northern muriquis [54]. No large fruit feeding-patch was available at the hill site, where fruit consumption thus required longer travel distances. With less time devoted to traveling, VG spent more time resting while camping out near fruit sources. This was true during the late dry season-early rainy season months of this study. Comparisons over a complete annual cycle would be necessary to evaluate whether the effects of microhabitats on howler behavior persist year-round, particularly later in the rainy season, when food resources are likely to be more abundant in both microhabitats [41,55]. On the other hand, as pointed out by Terborgh [56], it is in the dry season that important differences in foraging can be observed in primates; in times of plenty, all primates have very similar diets.

Differences in fruit production in both habitats may explain the dissimilarities found in the behavior of howlers in our study site since the VG had access to a number of large trees with a large fruit production within its home range. The hillside howlers inhabited a lower quality habitat in terms of structural and floristic aspects and the availability of preferred fruits, as indicated by their more folivorous diet.

These findings raise the question of how different groups secure their home ranges. If some home ranges are of higher quality than others, home range sites should become the object of contest competition between groups. The result would be a hierarchical ordering of groups in the forest, with higher-ranking groups securing better quality habitats. To date, such higher-level organization has not yet been studied in howler monkeys.

## Supporting Information

S1 Table Dietary composition of Valley and Hill groups. (DOCX)

S1 Fig Location of the study area and home range of the two study groups. (TIFF)
