# Supplementary material for: Effects of Local Habitat Variation on the Behavioral Ecology of Two Sympatric Groups of Brown Howler Monkey (Alouatta clamitans)
Source: PLoS One. 2015 Jul 6;10(7):e0129789. doi: 10.1371/journal.pone.0129789 (PMC4492992; doi:10.1371/journal.pone.0129789)
Supplement: S1 Table — (DOCX) [file pone.0129789.s001.docx]

**S1 Table. Supporting information –** Effects of Local Habitat Variation on the Behavioral Ecology of Two Sympatric Groups of Brown Howler Monkeys (*Alouatta clamitans*)

Linda Jung, Italo Mourthe, Carlos E. V. Grelle, Karen B. Strier & Jean P. Boubli^*^

^*^ Correspondence author: j.p.boubli@salford.ac.uk

**Table S1.** Dietary composition of Valley Group (VG; *n* = 184 feeding records) and Hill Group (HG; *n* = 316 feeding records).

| **Valley Group** | | | | | | | | | |
| --- | --- | --- | --- | --- | --- | --- | --- | --- | --- |
| **Family** | **Species** | **Fruit (%)** | **Mature leaf (%)** | **Immature leaf (%)** | **Unidentified leaf (%)** | **Flower (%)** | **Stem (%)** | **Unknow item (%)** | **Total (%)** |
| Annonaceae | *Xylopia* sp. | 0.1 | 0.0 | 0.0 | 0.0 | 0.0 | 0.0 | 0.0 | 0.1 |
| Bignoniaceae | *Tabebuia cassinoides* | 0.0 | 0.0 | 0.4 | 0.0 | 0.5 | 0.0 | 0.0 | 0.9 |
| Bignoniaceae | *Tabebuia caraiba* | 0.0 | 0.1 | 0.0 | 0.0 | 0.0 | 0.0 | 0.0 | 0.1 |
| Bignoniaceae | *Tabebuia serratifolia* | 0.0 | 0.3 | 0.0 | 0.0 | 0.0 | 0.0 | 0.0 | 0.3 |
| Bignoniaceae | *Tabebuia roseo-alba* | 0.0 | 0.6 | 0.0 | 0.0 | 0.0 | 0.0 | 0.1 | 0.7 |
| Bombacaceae | *Chorisia speciosa* | 0.0 | 0.0 | 0.1 | 0.0 | 0.0 | 0.0 | 0.0 | 0.1 |
| Euphorbiaceae | *Pogonophora schomburgkiana* | 0.0 | 0.1 | 0.0 | 0.0 | 0.0 | 0.0 | 0.0 | 0.1 |
| Flacourtiaceae | *Casearia ulmifolia* | 0.0 | 0.1 | 3.5 | 0.0 | 0.2 | 0.3 | 0.1 | 4.1 |
| Lauraceae | *Nectandra* sp. | 0.0 | 0.0 | 0.7 | 0.0 | 0.0 | 0.0 | 0.0 | 0.7 |
| Lauraceae | *Nectandra megapotamica* | 0.0 | 0.3 | 0.0 | 0.0 | 0.0 | 0.0 | 0.0 | 0.3 |
| Lauraceae | *Nectandra rigida* | 0.0 | 0.1 | 0.0 | 0.0 | 0.0 | 0.0 | 0.0 | 0.1 |
| Lecythidaceae | *Cariniana brasiliensis* | 0.0 | 0.3 | 0.0 | 0.0 | 0.5 | 0.0 | 0.0 | 0.8 |
| Lecythidaceae | *Lecythis* sp. | 0.0 | 0.9 | 0.5 | 0.0 | 0.0 | 0.0 | 0.1 | 1.4 |
| Leg./Caesalpinaceae | *Caesalpinia ferrea* | 0.0 | 3.0 | 0.3 | 0.0 | 0.0 | 0.0 | 0.0 | 3.3 |
| Leg./Caesalpinoideae | *Ocotea pretiosa* | 0.0 | 0.0 | 0.1 | 0.0 | 0.1 | 0.0 | 0.0 | 0.2 |
| Leg./Caesalpinoideae | *Apuleia leiocarpa* | 0.0 | 0.3 | 2.0 | 0.0 | 0.1 | 0.0 | 0.0 | 2.4 |
| Leg./Mimosoideae | *Peltophorum dubium* | 0.0 | 0.5 | 0.0 | 0.0 | 0.0 | 0.0 | 0.0 | 0.5 |
| Leg./Mimosoideae | *Piptadenia communis* | 0.0 | 0.2 | 0.0 | 0.0 | 0.0 | 0.0 | 0.0 | 0.2 |
| Leg./Mimosoideae | *Plathymenia foliolosa* | 0.0 | 0.0 | 0.2 | 0.0 | 0.0 | 0.0 | 0.0 | 0.2 |
| Leg./Papilionoideae | *Dalbergia nigra* | 0.0 | 0.3 | 1.1 | 0.0 | 0.0 | 0.0 | 0.0 | 1.4 |
| Leg./Papilionoideae | *Balfourodendron riedelianum* | 0.0 | 0.1 | 0.2 | 0.0 | 0.0 | 0.0 | 0.0 | 0.3 |
| Leg./Papilionoideae | Unidentified | 0.0 | 0.6 | 0.0 | 0.0 | 0.0 | 0.0 | 0.0 | 0.6 |
| Moraceae | *Ficus* sp. | 8.7 | 0.2 | 6.6 | 0.0 | 0.0 | 0.0 | 0.1 | 15.6 |
| Moraceae | *Acanthinophyllum ilicfolium* | 1.8 | 0.0 | 4.0 | 0.0 | 0.6 | 0.0 | 0.0 | 6.4 |
| Rutaceae | *Zanthoxylum rhoifolium* | 0.0 | 0.3 | 1.7 | 0.0 | 0.0 | 0.0 | 0.0 | 2.1 |
| Sapindaceae | *Allophylus edulis* | 0.0 | 1.2 | 0.9 | 0.0 | 0.0 | 0.0 | 0.0 | 2.1 |
| siehe canela sassafras | *Plathypodium elegans* | 0.0 | 0.0 | 0.2 | 0.0 | 0.0 | 0.0 | 0.0 | 0.2 |
| - | Lianas and vines | 0.0 | 1.8 | 0.1 | 0.0 | 1.9 | 0.2 | 0.0 | 4.0 |
| - | Unidentified | 4.3 | 22.2 | 14.1 | 0.8 | 2.5 | 0.4 | 6.4 | 50.7 |
| **Total** |  | 14.9 | 33.5 | 36.7 | 0.8 | 6.4 | 0.9 | 6.7 | 100.0 |
| **Hill Group** | | | | | | | | | |
| **Family** | **Species** | **Fruit (%)** | **Mature leaf (%)** | **Immature leaf (%)** | **Unidentified leaf (%)** | **Flower (%)** | **Stem (%)** | **Unknow item (%)** | **Total (%)** |
| Annonaceae | *Xylopia* sp. | 0.0 | 0.3 | 0.0 | 0.0 | 0.0 | 0.0 | 0.0 | 0.3 |
| Bignoniaceae | *Jacaranda copaia* | 0.0 | 0.2 | 0.0 | 0.0 | 0.0 | 0.0 | 0.0 | 0.2 |
| Dalbergieae | *Platypodium elegans* | 0.0 | 0.5 | 0.0 | 0.0 | 0.0 | 0.0 | 0.0 | 0.5 |
| Euphorbiaceae | *Pogonophora schomburgkiana* | 0.0 | 2.8 | 0.0 | 0.0 | 0.2 | 0.0 | 0.0 | 3.1 |
| Flacourtiaceae | *Casearia arborea* | 0.0 | 0.0 | 0.6 | 0.0 | 0.0 | 0.0 | 0.0 | 0.6 |
| Leg./Caesalpinaceae | *Caesalpinia ferrea* | 0.0 | 0.2 | 1.0 | 0.0 | 0.0 | 0.0 | 0.0 | 1.3 |
| Leg./Caesalpinoideae | *Plathypodium elegans* | 0.0 | 4.5 | 0.4 | 0.0 | 3.0 | 0.1 | 0.0 | 8.0 |
| Leg./Caesalpinoideae | *Apuleia leiocarpa* | 0.0 | 6.3 | 13.5 | 0.0 | 2.8 | 0.3 | 0.5 | 23.3 |
| Leg./Caesalpinoideae | *Schlerolobium parahyba* | 0.0 | 0.6 | 0.7 | 0.0 | 0.0 | 0.1 | 0.0 | 1.4 |
| Leg./Mimosoideae | *Anadenanthera colubrina* | 0.0 | 1.9 | 0.3 | 0.0 | 0.0 | 0.0 | 0.0 | 2.2 |
| Leg./Mimosoideae | *Peltophorum dubium* | 0.0 | 1.5 | 0.0 | 0.0 | 0.0 | 0.2 | 0.0 | 1.6 |
| Leg./Mimosoideae | *Piptadenia communis* | 0.0 | 0.0 | 0.3 | 0.0 | 0.0 | 0.0 | 0.0 | 0.4 |
| Leg./Mimosoideae | *Anadenanthera peregrina* | 0.0 | 5.2 | 0.0 | 0.0 | 0.0 | 0.0 | 0.0 | 5.2 |
| Leg./Mimosoideae | *Piptadenia gonoacantha* | 0.0 | 1.1 | 0.3 | 0.0 | 0.0 | 0.0 | 0.0 | 1.4 |
| Leg./Mimosoideae | *Plathymenia foliolosa* | 0.0 | 0.3 | 0.0 | 0.0 | 0.0 | 0.0 | 0.0 | 0.3 |
| Leg./Papilionoideae | *Vataereopsis araroba* | 0.0 | 0.2 | 0.0 | 0.0 | 0.0 | 0.0 | 0.0 | 0.2 |
| Leg./Papilionoideae | *Machaerium hirtum* | 0.0 | 0.0 | 0.4 | 0.0 | 0.0 | 0.0 | 0.0 | 0.4 |
| Leg./Papilionoideae | *Dalbergia nigra* | 0.0 | 2.0 | 0.4 | 0.0 | 0.0 | 0.0 | 0.0 | 2.4 |
| Moraceae | *Ficus* sp. | 0.0 | 0.1 | 0.6 | 0.0 | 0.3 | 0.0 | 0.0 | 1.1 |
| Moraceae | *Acanthinophyllum ilicfolium* | 0.2 | 0.0 | 0.1 | 0.0 | 0.0 | 0.0 | 0.0 | 0.3 |
| Sapindaceae | *Allophylus edulis* | 0.0 | 0.3 | 0.0 | 0.0 | 0.0 | 0.0 | 0.0 | 0.3 |
| Tiliaceae | *Luehea grandiflora* | 0.0 | 0.4 | 0.1 | 0.0 | 0.0 | 0.0 | 0.0 | 0.5 |
| - | Lianas and vines | 0.0 | 0.8 | 0.5 | 0.0 | 0.0 | 0.0 | 0.0 | 1.3 |
| - | Unidentified | 2.8 | 15.8 | 13.1 | 0.1 | 4.5 | 3.5 | 4.0 | 43.7 |
| **Total** |  | 3.0 | 45.1 | 32.3 | 0.1 | 10.9 | 4.1 | 4.5 | 100.0 |
