# Supplementary figures and images for: Effects of Local Habitat Variation on the Behavioral Ecology of Two Sympatric Groups of Brown Howler Monkey (Alouatta clamitans)
Source: PLoS One. 2015 Jul 6;10(7):e0129789. doi: 10.1371/journal.pone.0129789 (PMC4492992; doi:10.1371/journal.pone.0129789)

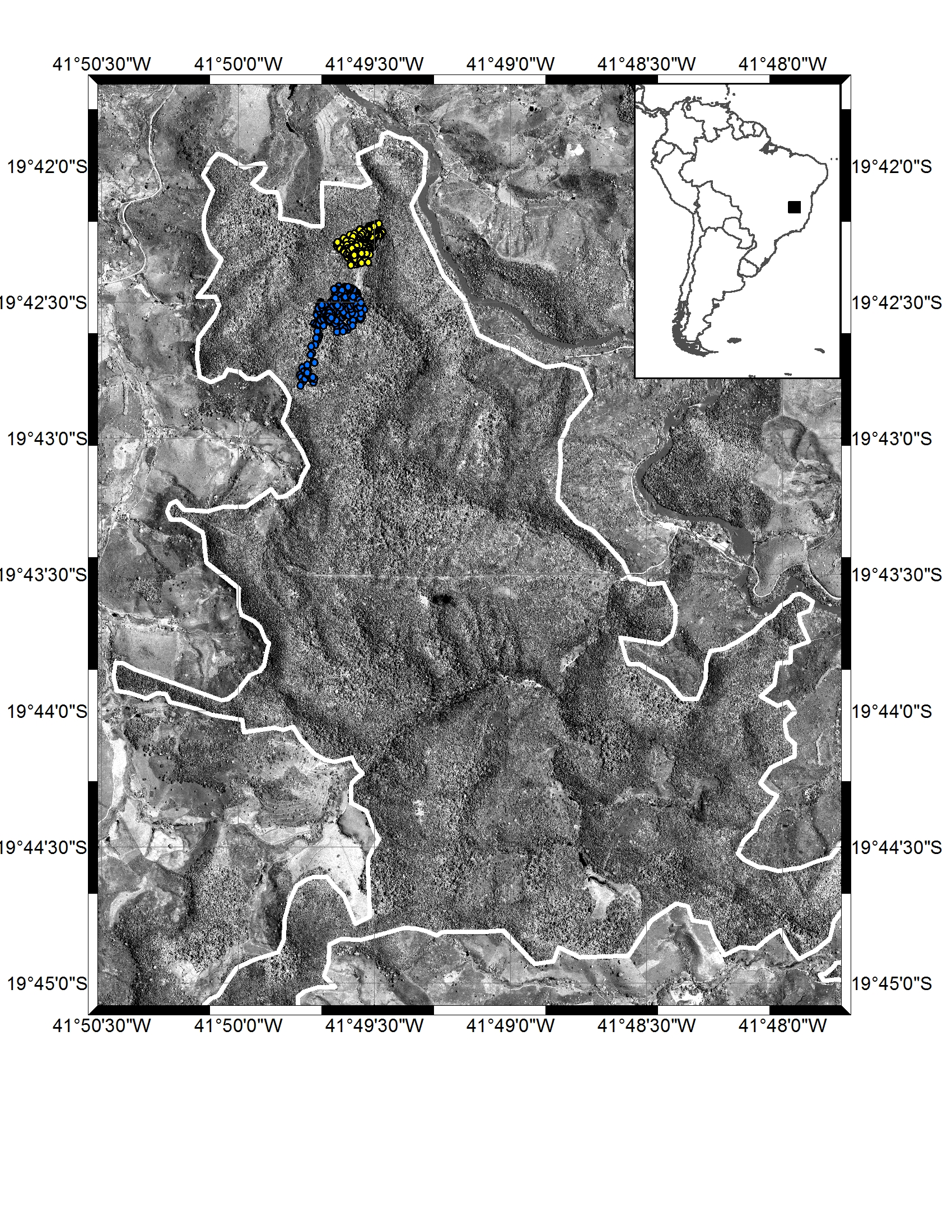

Supplement: S1 Fig — (TIFF) [file pone.0129789.s002.tiff]
